# Heat Shock Protein 60 Restricts Release of Mitochondrial dsRNA to Suppress Hepatic Inflammation and Ameliorate Non-Alcoholic Fatty Liver Disease in Mice

**DOI:** 10.3390/ijms23010577

**Published:** 2022-01-05

**Authors:** Ying-Hsien Huang, Feng-Sheng Wang, Pei-Wen Wang, Hung-Yu Lin, Sheng-Dean Luo, Ya-Ling Yang

**Affiliations:** 1 Department of Pediatrics, Kaohsiung Chang Gung Memorial Hospital Chang, Kaohsiung 833, Taiwan; yhhuang123@yahoo.com.tw; 2 College of Medicine, Gung University, Taoyuan 333, Taiwan; 3 Center for Mitochondrial Research and Medicine, Kaohsiung Chang Gung Memorial Hospital, Kaohsiung 833, Taiwan; wangfs@ms33.hinet.net; 4 Core Laboratory for Phenomics & Diagnostics, Department of Medical Research, Kaohsiung Chang Gung Memorial Hospital, College of Medicine, Chang Gung University, Kaohsiung 833, Taiwan; wangpw@adm.cgmh.org.tw; 5 Department of Internal Medicine, Kaohsiung Chang Gung Memorial Hospital, College of Medicine, Chang Gung University, Kaohsiung 833, Taiwan; 6 Research Assistant Center, Show Chwan Memorial Hospital, Changhua 500, Taiwan; linhungyu700218@gmail.com; 7 Department of Otolaryngology, Kaohsiung Chang Gung Memorial Hospital, College of Medicine, Chang Gung University, Kaohsiung 833, Taiwan; rsd0323@cgmh.org.tw; 8 Graduate Institute of Clinical Medical Sciences, College of Medicine, Chang Gung University, Taoyuan 333, Taiwan; 9 Department of Anesthesiology, Kaohsiung Chang Gung Memorial Hospital, College of Medicine, Chang Gung University, Kaohsiung 833, Taiwan

**Keywords:** HSP60, NAFLD, NASH, mitochondria, dsRNA, liver

## Abstract

Non-alcoholic fatty liver disease (NAFLD), the most common cause of chronic liver disease, consists of fat deposited (steatosis) in the liver due to causes besides excessive alcohol use. The folding activity of heat shock protein 60 (HSP60) has been shown to protect mitochondria from proteotoxicity under various types of stress. In this study, we investigated whether HSP60 could ameliorate experimental high-fat diet (HFD)-induced obesity and hepatitis and explored the potential mechanism in mice. The results uncovered that HSP60 gain not only alleviated HFD-induced body weight gain, fat accumulation, and hepatocellular steatosis, but also glucose tolerance and insulin resistance according to intraperitoneal glucose tolerance testing and insulin tolerance testing in HSP60 transgenic (HSP60Tg) compared to wild-type (WT) mice by HFD. Furthermore, overexpression of HSP60 in the HFD group resulted in inhibited release of mitochondrial dsRNA (mt-dsRNA) compared to WT mice. In addition, overexpression of HSP60 also inhibited the activation of toll-like receptor 3 (TLR3), melanoma differentiation-associated gene 5 (MDA5), and phosphorylated-interferon regulatory factor 3 (p-IRF3), as well as inflammatory biomarkers such as mRNA of il-1β and il-6 expression in the liver in response to HFD. The in vitro study also confirmed that the addition of HSP-60 mimics in HepG2 cells led to upregulated expression level of HSP60 and restricted release of mt-dsRNA, as well as downregulated expression levels of TLR3, MDA5, and pIRF3. This study provides novel insight into a hepatoprotective effect, whereby HSP60 inhibits the release of dsRNA to repress the TLR3/MDA5/pIRF3 pathway in the context of NAFLD or hepatic inflammation. Therefore, HSP60 may serve as a possible therapeutic target for improving NAFLD.

## 1. Introduction

Non-alcoholic fatty liver disease (NAFLD), the most common cause of chronic liver disease, consists of fat deposited (steatosis) in the liver due to causes other than excessive alcohol use [1]. Western countries have a high prevalence of NAFLD, which increases in parallel with the incidence of obesity, diabetes, and aging [1]. Meanwhile, a significant increased prevalence of NAFLD has also been observed in Taiwan in recent decades [2]. NAFLD is a hepatic manifestation of the metabolic syndrome [3]. Notably, NAFLD is also closely associated with cardio- and neurological complications, including cardiovascular disease, hypertension, cognitive dysfunction, and ischemic stroke [4,5]. NAFLD constitutes a spectrum of liver disorders that begins with simple steatosis and can then progress to more extreme conditions, such as non-alcoholic steatohepatitis (NASH), non-alcoholic steatofibrosis (NASF), cirrhosis, and hepatocellular carcinoma (HCC) [6].

In the pathogenesis of fatty liver disease, the concurrent reduced levels of β-oxidation, along with increased lipogenesis, result in lipid accumulation in hepatocytes, as well as the subsequent production of reactive oxygen species (ROS) and hepatocyte injury, both of which contribute to activation of both Kupffer cells (KC) and hepatic stellate cells (HSC) [7]. Mitochondrial dysfunction combined with the release of mitochondrial damage-associated molecular patterns (mt-DAMP) is key to HSC activation and liver fibrosis [8]. On the other hand, mitochondrial quality control mechanisms, including mitochondrial unfolded protein response (UPR^mt^), dynamics, and mitophagy, are central to maintaining mitochondrial homeostasis [9,10]. UPR^mt^ constitutes mito-nuclear communication of stress signals derived from mitochondria-promoting transcriptional activity in the nucleus that generates mitochondrial chaperones (e.g., heat shock protein 10, 60, and 90 (HSP10, 60, and 90)) and proteases for repairing unfolded protein stress [11].

HSP60, also known as HSPD1, is a 60 kDa chaperone primarily found in the mitochondrial matrix [12]. The folding activity of HSP60 protects mitochondria from proteotoxicity under various types of stress [13,14,15]. The loss of HSP60 in cardiomyocytes results in impaired mitochondrial membrane potential and respiratory enzyme activity, as well as an increase in reactive oxygen species (ROS) production [16]. Mice with heterozygous knockout of HSP60 presence impaired folding of manganese superoxide dismutase, which is a mitochondrial antioxidant enzyme, and demonstrated oxidative stress in neuronal tissues [17]. Our recent studies revealed that the overexpression of microRNA-29a (miR-29a) caused NAFLD and NASH improvement [18,19,20,21,22] and that miR-29a was associated with upregulated HSP60 [23], implying that HSP60 may play a role in treating NAFLD and repression hepatic inflammation. Dhir et al. identified the dsRNA of mitochondrial origin (mt-dsRNA) as a critical mt-DAMP, which plays a critical role in triggering melanoma differentiation-associated protein 5/mitochondrial antiviral signaling protein/interferon-stimulated genes (MDA5/MAVS/ISGs)-related innate immune signaling [24]. Furthermore, Lee et al. discovered that mt-dsRNA engulfed by exosomes is implicated in provoking the pro-inflammatory activity of KC in an alcoholic liver disease model [25]. As HSP60 possesses an emerging role in modulating inflammatory conditions, whether its folding activity is implicated in mt-dsRNA restriction in the context of NAFLD remains unanswered. In this study, we have demonstrated that overexpression of HSP60 ameliorates a high-fat diet (HFD)-induced release of mt-dsRNA to suppress hepatic inflammation and improves glucose intolerance and insulin resistance in an animal model of NAFLD.

## 2. Results

### 2.1. Overexpression of HSP60 Significantly Reduces Weight Gain and Fat Accumulation in Adipose Tissue and Improves Glucose Tolerance and Insulin Resistance

**Figure 1:**
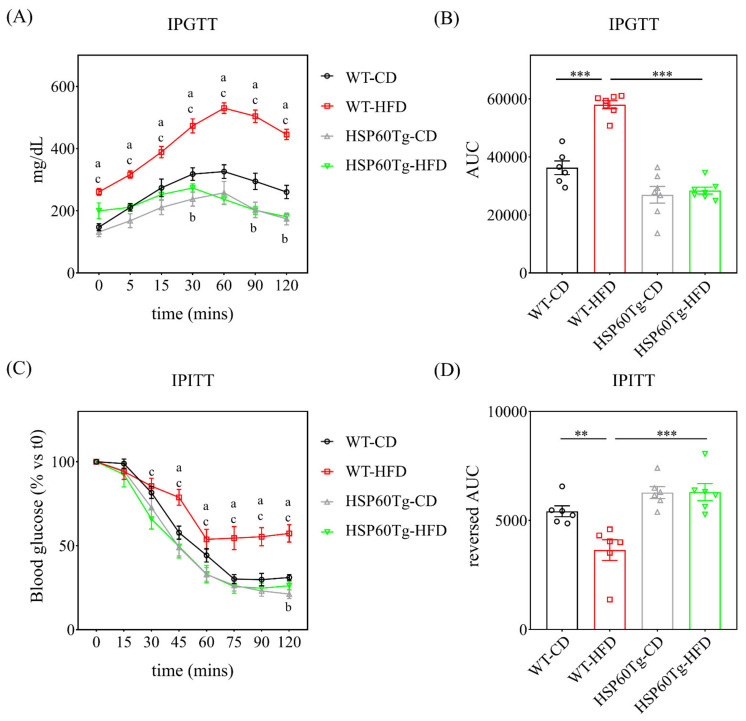
Comparison of IPGTT and IPITT among the different groups. Both tests were performed on mice at the end of the 6-month period of CD or HFD. (**A**) IPGTT was analyzed by the corresponding (**B**) AUC obtained from changes in plasma glucose. (**C**) IPITT was analyzed by corresponding with the reverse (**D**) AUC data obtained. Results were obtained from six to seven independent experiments and data expressed as the mean ± standard error. ^a^ *p* < 0.05 WT-CD versus WT-HFD; ^b^ *p* < 0.05 WT-CD versus HSP60Tg-CD; ^c^ *p* < 0.05 WT-HFD versus HSP60Tg-HFD. Intraperitoneal glucose tolerance test (IPGTT) and insulin tolerance test (ITT); chow diet (CD); high-fat diet (HFD); area under curve (AUC); transgenic heat shock protein 60 (HSP60Tg); wild type (WT). ** indicates *p* < 0.01, *** indicates *p* < 0.001.

To better understand the potential involvement of HSP60 in the development of NAFLD, we utilized a HFD composed of 60% calories from fat to induce an obesity animal model [19], which has been shown to induce steatosis and hepatic inflammation. Both wild-type (WT) and HSP60 transgenic (HSP60Tg) mice that were fed HFD showed significant weight gain (both *p* < 0.05, Table 1). However, the HSP60Tg-HFD mice had significantly less weight gain compared to the WT-HFD mice (*p* < 0.05, Table 1). Together with the weight gain, HFD also induced subcutaneous, visceral, and intestinal fat and liver weight in WT but only visceral fat in the HSP60 mice (Table 1). In addition, we observed no significant difference in subcutaneous and intestinal fat or liver weight of HSP60Tg-HFD and HSP60Tg-chow diet (CD). Moreover, the HSP60Tg-HFD mice presented lower subcutaneous, visceral, and intestinal fat and liver weight than the WT-HFD mice (all *p* < 0.05, Table 1). These results indicate that an overexpression of HSP60 may reduce fat accumulation and liver mass induced by HFD. In order to assay glucose tolerance and insulin resistance, we performed IPGTT and ITT in mice fed with chow and HFD for six months. As shown in Figure 1, compared with the control group, WT mice displayed glucose intolerance (*p* < 0.001) and insulin resistance (*p* < 0.01) after 6 six months of HFD. Interestingly, no significant difference in IPGTT and ITT was found in HSP60Tg mice after being fed with HFD compared to the CD. We further demonstrated that HSP60Tg mice also showed improved glucose tolerance and insulin sensitivity compared to WT mice after being fed with HFD (all *p* < 0.001).

### 2.2. Overexpression of HSP60 Reduces Hepatocellular Steatosis in the Context of Chronic HFD

**Figure 2:**
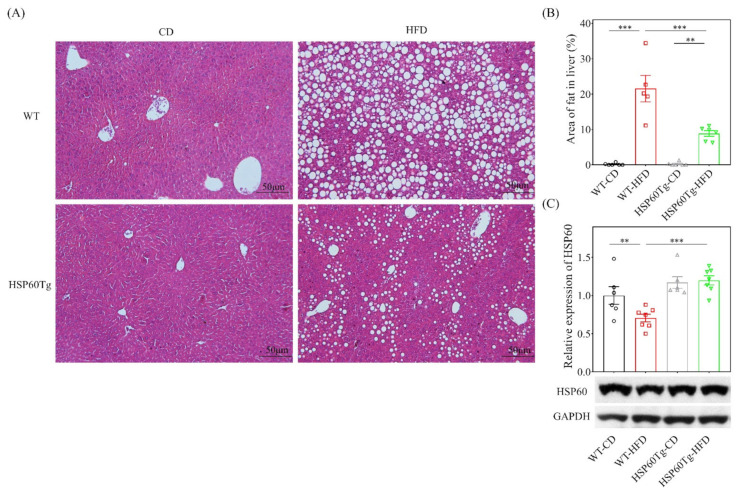
A gain of HSP60 reduces hepatocellular steatosis in the context of chronic HFD for 26 weeks. Paraformaldehyde-fixed paraffin-embedded liver tissue was used to determine the abundance of lipid droplets with hematoxylin–eosin (HE) stain. (**A**) Representative HE stains image of each group. (**B**) Lipid droplet area quantified using ImageJ. (**C**) The expression of HSP60 in tissues from the HFD group was significantly lower than in tissues from the WT mice. Furthermore, overexpression of HSP60 was significantly greater in HSP60Tg-HFD mice compared with the WT-HFD group. Data collected from six fields of view of each specimen and five to seven specimens for each group are expressed as the mean ± SE. ** *p* < 0.01 and *** *p* < 0.001 between the indicated groups. WT, wild-type mice; HFD, high-fat diet; HSP60Tg, mice harboring overexpression of heat shock protein 60; CD, chow diet.

Next, we examined the hepatic steatosis status of the liver. An abundance of lipid droplets was assayed using hematoxylin and eosin stain. As shown in Figure 2, HFD led to significantly increased fat accumulation in the liver in both WT and HSP60 Tg mice (*p* < 0.001 in Figure 2A,B). Of particular note, HSP60Tg-HFD mice expressed a significantly lower droplet count percentage than WT-HFD mice (*p* < 0.001, Figure 2A,B). We also used Western blotting to measure the HSP60 expression after mice were fed with HFD. The expression level of HSP60 was downregulated in WT mice after being fed with HFD (Figure 2C, *p* < 0.01). HSP60Tg mice showed a higher level of HSP60 than WT mice after being fed with HFD (Figure 2C, *p* < 0.001). This result indicates that overexpression of HSP60 effectively improved hepatocellular steatosis induced by HFD.

### 2.3. Overexpression of HSP60 Represses the Release of mt-dsRNA and Inflammation in the Context of Chronic HFD

**Figure 3:**
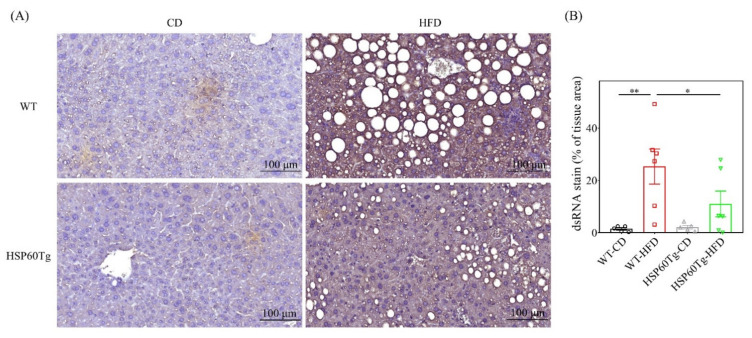
A gain of HSP60 reduces mt-dsRNA in the context of chronic HFD for 26 weeks. Paraformaldehyde-fixed paraffin-embedded liver tissue was used to determine the mt-dsRNA using immunofluorescence staining. (**A**) Representative stain image of each group. Brown color indicates positive signal of mt-dsRNA. (**B**) Positive signal percentage quantified using ImageJ. Data collected from six fields of view of each specimen and five to six specimens for each group are expressed as the mean ± SE. * *p* < 0.05 and ** *p* < 0.01 between the indicated groups. WT, wild-type mice; HFD, high-fat diet; HSP60Tg, mice harboring overexpression of heat shock protein 60; CD, chow diet; mt-dsRNA, mitochondria double-strain RNA.

**Figure 4:**
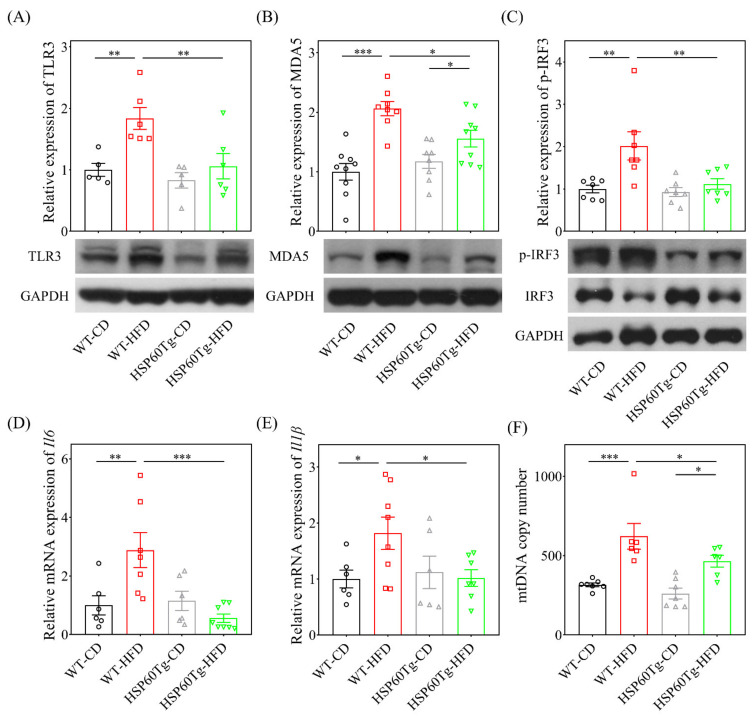
Analyses of the protein of TLR3 (**A**), MDA5 (**B**), and p-IRF3 (**C**), the mRNA of the il-6 (**D**) and il1-il1β (**E**) expressions, and the (**F**) mitochondria DNA copy number per cell in the WT and HSP60Tg mice livers following HFD or CD for 26 weeks. Data calculated from five to eight samples per group are expressed as the mean ± SE. * *p* < 0.05, ** *p* < 0.01 and *** *p* < 0.001 between the indicated groups. WT, wild-type mice; HFD, high-fat diet; HSP60Tg, mice harboring overexpression of heat shock protein 60; CD, chow diet; TLR3, toll-like receptor 3; MDA5, melanoma differentiation-associated gene 5; p-IRF3, phosphorylated-interferon regulatory factor 3; GAPDH, glyceraldehyde 3-phosphate dehydrogenase.

We then investigated whether HSP60 modulates the release of mt-dsRNA and inflammation in HFD-induced liver injury. As shown in Figure 3, HFD induces a significantly increased expression level of mt-dsRNA in WT mice (*p* < 0.001), but not in HSP60Tg mice (*p* = 0.191). Notably, HSP60Tg-HFD mice demonstrated a significantly reduced release of mt-dsRNA compared to WT-HFD mice (*p* < 0.05, Figure 3). Next, we studied the downstream inflammatory markers of mt-dsRNA. We observed that the expression levels of TLR3, MDA5, and p-IRF3 protein were all decreased, as were the il6 and il1β mRNA levels, in the liver of HSP60Tg-HFD compared to WT-HFD mice (*p* < 0.01, *p* < 0.05, *p* < 0.01, *p* < 0.01 and *p* < 0.05, respectively, Figure 4A–E). This finding suggests that HSP60 overexpression exerts a suppressive effect on mt-dsRNA/TLR3/MDA5 inflammatory signal pathways in the HFD-induced hepatic steatohepatitis process.

### 2.4. Overexpression of HSP60 Modulates HFD-Caused Perturbation of Mitochondrial DNA Content in the Liver

In our previous study, we found significantly high levels of mitochondria DNA (mtDNA) content in the liver of WT-HFD mice [19]. As a result, in this study, we looked for a link between HSP60 and mitochondrial biogenesis. The MtDNA content was detected using qPCR. As shown in Figure 4F, HFD significantly increased the mtDNA content in the liver of WT mice (*p* < 0.001). HSP60Tg-HFD mice exhibited a significantly lower level of mtDNA content in the liver than WT-HFD mice (*p* < 0.05). These data reveal that HSP60 overexpression decreases HFD-elicited and mtDNA content during hepatosteatosis.

### 2.5. HSP60 Restricts the Release of Mitochondrial dsRNA In Vitro

**Figure 5:**
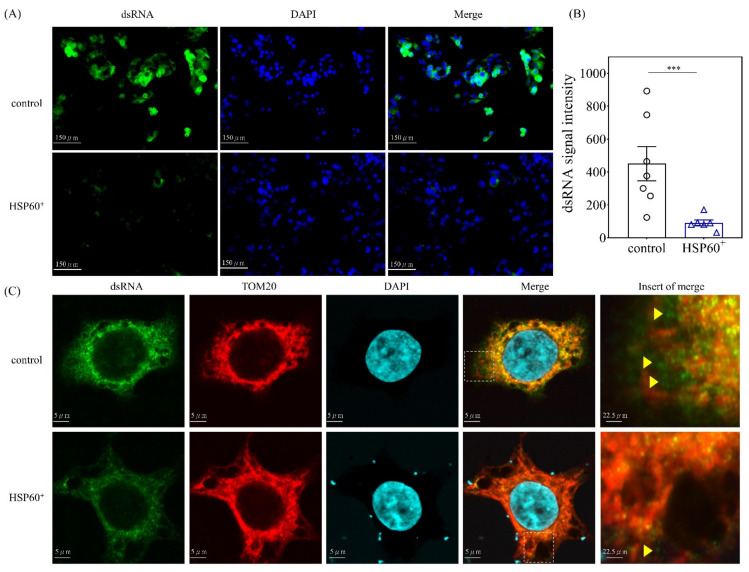
(**A**) Immunofluorescence analysis of mt-dsRNA in HepG2 cells after treatment with pUSE-amp vector containing HSP60 for 24 h. The mt-dsRNA levels are significantly lower in containing the HSP60-transfected vector in vitro. (**B**) Data are expressed as the mean ± SE of the six to seven samples per group. *** indicates a *p* < 0.001 (**C**) Tom20 is the mitochondrial outer membrane protein. Confocal image study confirmed that HSP60 restricts the release of mt-dsRNA (green) in vitro.

Next, we studied whether HSP60 could restrict the release of mt-dsRNA in vitro. We first examined the mt-dsRNA expression in HepG2 cells. As shown in Figure 5A,B, the mt-dsRNA levels were significantly lower in the pUSE-amp vector containing HSP60-transfected cell cultures (*p* < 0.001). Tom20 is the mitochondrial outer membrane protein [26]. As shown in Figure 5C, the confocal image study confirmed that HSP60 restricts the release of mt-dsRNA (green) in vitro.

### 2.6. Gain of HSP60 Suppresses TLR3 and MDA5 In Vitro

**Figure 6:**
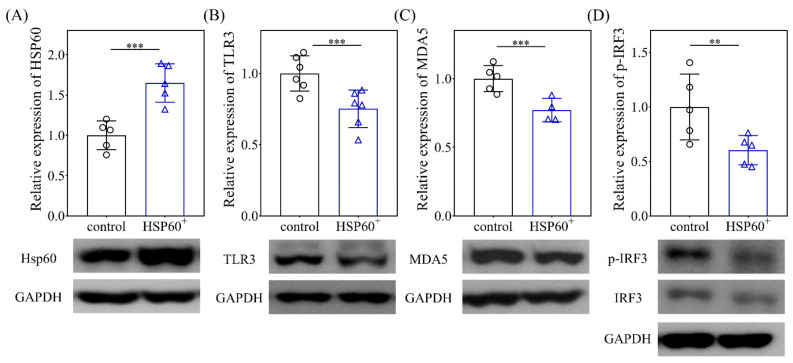
Analyses of HSP60 (**A**), TLR3 (**B**), MDA5 (**C**), and p-IRF3 (**D**) on the 10th culture day of HepG2 after treatment with a HSP60 overexpression vector for 24 h. Data are expressed as the mean ± SE of the four to six samples per group. ** *p* < 0.01 and *** *p* < 0.001 between the indicated groups. TLR3, toll-like receptor 3; MDA5, melanoma differentiation-associated gene 5; P-IRF3, phosphorylated-interferon regulatory factor 3; GAPDH, glyceraldehyde 3-phosphate dehydrogenase.

We then evaluated the effect of overexpression of HSP60 mimics on the behavior of the HCC cell line. As shown in Figure 6, the addition of HSP60 mimics induced a significant increase in the protein level of HSP60 and a decrease in TLR3, MDA5, and p-IRF3 protein levels (*p* < 0.001, *p* < 0.001, *p* < 0.001, *p* < 0.001, *p* < 0.01, respectively), which is compatible with the observations of our animal model.

## 3. Discussion

Numerous studies have clearly illustrated that NAFLD is a systemic metabolic disorder [21]. As a result, various papers have taken the initiative to propose a new name: metabolic-associated fatty liver disease (MAFLD) and metabolic-associated steatohepatitis (MASH) [27,28,29]. The criteria for MAFLD requires evidence of hepatic steatosis accompanied by at least one of the following three features: overweight/obesity, type 2 diabetes mellitus (T2DM), or evidence of metabolic dysregulation [30]. In this study, we revealed the regulatory role of HSP60 in mitigating NAFLD, as well as the improved glucose tolerance and insulin resistance in long-term HFD in an animal model. Furthermore, HSP60 overexpression in a HFD condition exerts a repressive effect on proinflammatory cytokine IL-6 and IL-1β, as well as on the GPT levels and mt-dsRNA/TLR3/MDA5 signaling pathways, all of which are implicated in steatohepatitis. Furthermore, HSP60 suppresses HFD-induced mtDNA copy number, which has been shown to be increased in the liver [19] and associated with increased oxidative damage and inflammation in adipose tissue of mice that have been fed HFD [31].

mt-dsRNA is a critical mt-DAMP, and under some pathophysiological conditions, mt-dsRNA released to the cytoplasm is implicated in triggering innate immune responses [32] and eliciting the pro-inflammatory activity of KC in an alcoholic liver disease model [25]. The released mt-dsRNA moves from hepatocyte to KC and triggers pro-inflammatory signaling in a TLR3-dependent manner [25]. In addition, several studies have shown the immunomodulatory effect of HSP60 [33]. Exogenous HSP60 alleviates hepatic inflammation in an experimental hepatitis mouse model via a downregulated pro-inflammatory profile in T cells [34]. Recently, Tsai et al. discovered that HSP60 expression was decreased in HFD mice and could be rescued by N-acetylcysteine therapy [35]. Both intra-articular injection of exogenous HSP60 and transgenic HSP60 overexpression (HSP60Tg) demonstrate resistance against experimental joint inflammation compared to the mock control and wild-type (WT) groups [36]. Here, we demonstrated that mice harboring an overexpression of HSP60 presented suppressed mt-dsRNA abundance, as well as reduced body weight and adipose tissue of various parts, in line with the notion of suppressed TLR3/MDA5/p-IRF3 signaling pathways and pro-inflammatory cytokines expression. Recently, one study showed that TLR3 ablation prevented obesity and metabolic disorders [37].

**Figure 7:**
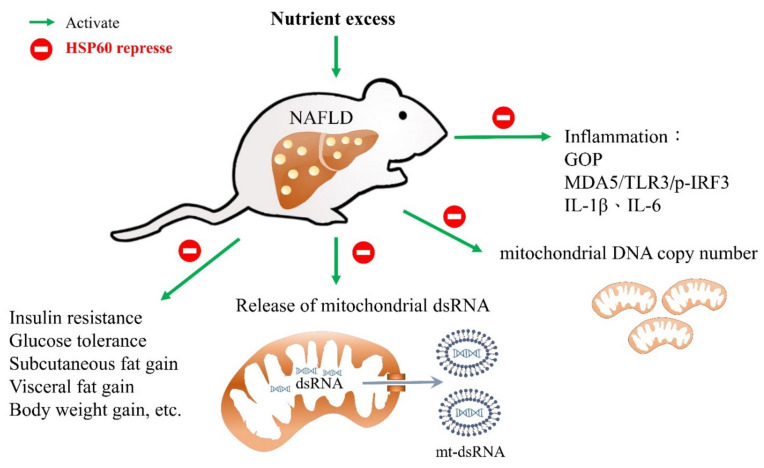
Proposed model of heat shock protein 60 restricts release of mitochondrial dsRNA to suppress hepatic inflammation and ameliorate non-alcoholic steatohepatitis in mice. High-fat diet (HFD) composed of 60% calories from fat can induce an obesity animal model that is characterized by steatohepatitis, glucose intolerance, and insulin resistance. Gain of heat shock protein 60 (HSP60) alleviated not only HFD-induced body weight gain, fat accumulation, and hepatocellular steatosis, but also glucose tolerance and insulin resistance. Furthermore, overexpression of HSP60 in the HFD group resulted in inhibited release of mitochondrial dsRNA (mt-dsRNA) compared to wild-type mice. In addition, overexpression of HSP60 also inhibited the activation of toll-like receptor 3 (TLR3), melanoma differentiation-associated gene 5 (MDA5), and phosphorylated-interferon regulatory factor 3 (p-IRF3), as well as inflammatory biomarkers such as mRNA of il-1β and il-6 expression in the liver in response to HFD.

MDA5 is known to be a member of retinoic acid-inducible gene-I-like receptors, as well as to play an important role in the inflammation of NASH [38]. Furthermore, activation of the stimulator of interferon genes and its downstream factor IRF3 can promote hepatocellular injury by inducing inflammatory cytokines and disturbing glucose and lipid metabolism [39]. Herein, HSP60 gain also improves hepatocellular inflammation, lipid, and glucose metabolism in response to HFD. Our novel findings of HSP60 and mt-dsRNA provide new insight into the crosstalk between lipid/glucose metabolism and inflammation in the progression of overnutrition-caused liver disease. The proposed schematics depicting the effect of HSP60 in counteracting HFD-induced liver steatosis is presented in Figure 7.

In this study, we conclude that the overexpression of HSP60 in HFD can ameliorate hepatocellular steatosis and liver inflammation, likely by targeting the release of mt-dsRNA and the subsequent innate immunity response, including a reduction in the TLR3/MDA5 signaling pathways, thus providing a potential target for modulating the NAFLD process. Furthermore, we also proposed an HSP60-based molecular approach for reducing obesity in an animal model. Nevertheless, further investigation is warranted to elaborate on the exact mechanism and clinical implication.

## 4. Materials and Methods

### 4.1. Ethics Statement and Animal Protocol

The animal protocol was reviewed and approved by the Institutional Animal Care and Use Committee (IACUC) of Chang Gung Memorial Hospital (Approval number: 2020031701). FVB/NJNarl mice weighing 25–35 g were purchased from a standardized SPF Laboratory (NLAC, Taipei, Taiwan). All animals were housed at the Center for Laboratory Animals of Kaohsiung Chang Gung Memorial Hospital in compliance with the Institutional Animal Care and Use Committee guidelines and were individually housed under controlled temperature (22 °C), humidity (55%), and lighting (12:12 h light:dark cycle), with free access to water and food. For the NAFLD model and corresponding normal control, eight-week-old male FVB/NJNarl mice of HSP60Tg and WT mice (N = 6–8) were fed with a HFD (58 kcal% fat and sucrose, D12331, OPENSOURCE) or chow diet for 26 weeks, respectively.

### 4.2. Construction and Breeding of the HSP60 Transgenic Mice

Transgenic (Tg) mice (FVB/NNarl-TgPGK-HSP60; TgHSP60) were generated by overexpressing human HSP60 driven by phosphoglycerate kinase (PGK) promoter, which were generated as previously described in another study [36]. Briefly, the linear PGK-HSP60-BGH-poly-A construct was cloned, purified, and sequence validated. Fertilized eggs harvested from FVB/N mice were microinjected (Zeiss) with the constructs under aseptic conditions and then transplanted to the institute of cancer research foster mothers accommodated in a specific pathogen-free vivarium. Genotypes of transgenic mice were validated using PCR protocols and primers (forward, 5-ATGCTTCGGTTACCCACAGTCTTTCGCCAGATGA′; reverse, 5′-TTAGAACATGCCACCTCCCATACCACCTCCCATT′). Age-matched male mice without the construct of interest were used as the wild-type (WT) control.

### 4.3. Blood Biochemical Analysis

Serum was preserved at −80 °C until the biochemical analysis was performed. The serum alanine aminotransferase (GPT), triglycerides, and total cholesterol level of the mice were analyzed with a SPOTCHEM EZ (SP-4430, Arkray, Kyoto, Japan) automated biochemical analyzer.

### 4.4. Assessment of IPITT and IPGTT

All animals fasted 16 h before experiments were performed. Mice were placed in restrainers, and blood samples were obtained by tail bleeding and then analyzed by a glucose meter (Optium Xceed XCN 289-2337; Abbott). For IPGTT, blood glucose was measured immediately before and at 5, 15, 30, 60, 90, and 120 min after an intraperitoneal glucose (1 g/kg in 0.9% NaCl) injection. For IPITT, blood glucose was measured immediately before and at 15, 30, 45, 60, 75, 90, and 120 min after an IP insulin (0.75 U/kg) injection.

### 4.5. Cell Culture and Transfection

Human HCC cell line HepG2 was cultured in DMEM medium supplemented with 10% heat-inactivated fetal bovine serum (FBS), glutamax, and antibiotic-antimycotic at 37 °C in a humidified incubator with 5% CO_2_. Cells were seeded at a density of 1.5 × 10^6^ cells per 6-cm culture dish for Western blotting and 1.5 × 10^5^ cells/well in the 12-well culture plate for dsRNA staining. Twenty-four hours after the initial seeding, we transfected the HepG2 cells with 3 μg HSP60 vector (pUSE-amp vector containing HSP60) using the Lipofectamine™ RNAiMAX Transfection Reagent (Invitrogen, Carlsbad, CA, USA) according to the manufacturer’s instructions. Infected cells were incubated for 24 h at 37 °C and then used for further experiments.

### 4.6. Hematoxylin and Eosin Stain Staining

For histological examination, liver tissues were preserved in 10% formaldehyde, embedded in paraffin, and cut into 2-μm-thick sections for hematoxylin and eosin staining. Hepatic steatosis was determined by quantification of the lipid droplets area. Images were photographed for six low-power fields per slide (magnification, ×40), as described in a previous study [40].

### 4.7. Immunohistochemistry and Immunofluorescence Staining

For immunohistochemistry staining, the liver tissues were preserved in 10% formaldehyde, embedded in paraffin, and cut into 2-μm-thick sections. After deparaffinization and rehydration, the sections were heated in a citrate buffer (10 mM, pH 6, Thermo, Waltham, MA, USA) in a microwave for 30 min to retrieve the antigens. We washed all sections with PBS and then stained them using the UltraVision Quanto Detection System HRP DAB kit (Thermo, Waltham, MA, USA) and hybridized with the anti-dsRNA antibody (1:200, JENA, Germany). Then the sections were counterstained with Mayer’s hematoxylin (ScyTek Laboratories, West Logan, UT, USA), dehydrated, and mounted using a sub-X mounting medium (3801740, Leica, IL, USA) according to the manufacturer’s instructions. For the quantification data, we used ImageJ to calculate the percentage of brown areas. For immunofluorescence staining, after treatment on glass coverslips (Thermo, Waltham, MA, USA), the cells were washed with PBS for 5 min, then fixed in 4% paraformaldehyde for 40 min at room temperature, washed twice with PBS for 5 min, and stored with PBS overnight at 4 °C. The cells were permeabilized with PBS containing 0.1% Triton X-100 for 20 min and washed with PBS three times for 3 min, then blocked with Ultra V Blocking buffer (TA-060-UB, Thermo, Waltham, MA, USA) for 30 min at room temperature. Afterward, the cells were washed with PBS for 5 min and incubated with dsRNA antibody (RNT-SCI-10030005, 1:200, JENA, Germany) for 2 h in a 37 °C oven, followed by washing with PBST three times for 5 min before incubation with other primary antibodies (TOM20, 1:200, 11802-1-AP, PROTEINTECH, IL, USA) for 1 h in a 37 °C oven and then washed with PBS three times for 5 min. Second antibodies (anti-rabbit-488, 1:500, anti-mouse-595, 1:500, abcam, Cambridge, UK) were incubated for 1 h in a 37 °C oven, then washed with PBST three times for 5 min, stained with DAPI (1:500) for 10 min in a 37 °C oven, and then washed with PBST twice for 3 min again. The stained cells were mounted with fluorescent mounting medium (F4680, Sigma, MA, USA), then visualized by microscopy (Olympus). We quantified the fluorescent intensities on independent color channels using ImageJ analysis.

### 4.8. RNA Isolation and Quantitative Real-Time PCR

Total RNA was extracted from the liver tissue by using TRIzol^®^ reagent (15596026, Invitrogen, Carlsbad, CA, USA) and was then submitted to reverse transcription to yield cDNA with an oligodeoxynucleotide primer (oligo dT15)-based method according to the manufacturer’s protocol (M1701, Promega, Madison, WI, USA). The qPCR reaction for *Il6* and *Il1β* and normalization control *Gapdh* were conducted with 2× SYBR Green PCR Master Mix (04887352001, Roche, CA, USA) on a LightCycler480^®^ (Roche). Each PCR reaction included 0.5 μM forward and reverse primers, 30 ng of cDNA, and 1× SYBR Green PCR Master Mix in a total reaction volume of 10 μL. The qPCR program included an initial denaturation step at 95 °C for 10 min, followed by 45 cycles of denaturation at 95 °C for 30 s, annealing at 62 °C for 15 s, and extension at 72 °C for 25 s, with a final step for melting curve analysis. The primers sequence was as follows: *Il6* forward 5′-GACAAAGCCAGAGTCCTTCAGA-3′, *Il6* reverse 5′-AGGAGAGCATTGGAAATTGGGG-3′; *Il1β* forward 5′-TAACCTGCTGGTGTGTGAC-3′, *Il1β* reverse sequence 5′-CATTGAGGTGGAGAGCTTTC-3′; *Gapdh* forward 5′-GCACAGTCAAGGCCGAGAAT-3′, *Gapdh* reverse sequence 5′-GCCTTCTCCATGGTGGTGG-3′. We based the calculation of relative gene expression on the comparative cycle threshold (CT) method, whereby the value of the target gene was given by 2^−(^^Δ^^CT target−^^Δ^^CT calibrator)^ or 2^−^^ΔΔ^^CT^.

### 4.9. Western Blot

For the Western blot test, we measured protein concentration using the Bio-Rad Protein Assay Reagent (Bio-Rad, Berkeley, CA, USA) and then mixed 30~60 μg protein with 4× sample buffer (Bio-Rad, Berkeley, CA, USA), boiled them for 10 min, and performed electrophoresis using 8~15% SDS-PAGE gels. Following electrophoresis, it was transferred to a PVDF membrane (Millipore, MA), and the blots were incubated with primary antibodies at 4 °C overnight against HSP60 (sc-1052, Santa Cruz, CA, USA), TLR3 (ab62566, abcam, Cambridge, UK), MDA5 (#5321, Cell Signaling, MA, USA), p-IRF3 (orb6225, Biorbyt, Cambridge, UK), IRF3 (ab68481, abcam, Cambridge, UK) and GAPDH (60004-1-lg, PROTEINTECH, IL, USA) for protein control. They were then washed with TBS-T (0.05% tween20), reacted with secondary polyclonal antibody against rabbit IgG (PerkinElmer, Waltham, MA, USA), mouse IgG (PerkinElmer, Waltham, MA, USA), and goat IgG antibodies at room temperature for 1 h, developed with enhanced chemiluminescence detection (GE Healthcare Biosciences AB, Uppsala), and exposed to film. The signals were quantified by volume using Quantity One^®^ 1-D analysis software (Bio-Rad Laboratories).

### 4.10. Quantification of mtDNA Copy Number by Real-Time PCR

mtDNA was extracted from mouse liver tissue using TOOLS Genomic DNA Extraction Kit (TOOLS, DPT-BC04 -02, NTPC, TW). The relative mtDNA copy numbers were measured using real-time PCR and corrected by simultaneous measurement of the nuclear DNA. The PCR was performed in a LightCycler480^®^ System (Roche), using the SYBR Green PCR Master Mix Kit (2×) (04887352001, Roche, CA, USA). The threshold cycle number (Ct) values of the *Tert* gene and the mitochondrial *ND1* gene were determined for each in the same quantitative PCR run. Each measurement was performed at least three times and normalized in each experiment against serial dilution of a control DNA sample. Ct values were used as a measure of the input copy number, and we used Ct value differences to quantify mtDNA copy number relatives. The primers sequence was as follows: *Nd1* forward 5′-ACCATTTGCAGACGCCATAA-3′, *Nd1* reverse 5′-TAAATTGTTTGGGCTACGG-3′; *Tert* forward 5′-CTAGCTCATGTGTCAAGACCCTCTT-3′, *Tert* reverse sequence 5′-GCCAGCACGTTTCTCTCGTT-3′.

### 4.11. Statistical Analysis

All values are expressed as the mean ± standard error. We used one-way analysis of variance (ANOVA) to analyze the quantitative data when appropriate. The Bonferroni test was used for post-hoc testing as necessary. Two-sided *p*-values less than 0.05 were considered statistically significant.

## Figures and Tables

**Table 1:** Comparison of the changes in the four groups.

	WT-CD	WT-HFD	HSP60Tg-CD	HSP60Tg-HFD
Body weight (g)	35.01 ± 0.65	46.74 ± 1.04 ^a^	30.26 ± 0.67 ^a^	33.87 ± 0.97 ^bc^
GPT (dU/L)	20.6 ± 6.1	58.1 ± 7.1 ^a^	16.7 ± 4.2	28.8 ± 7.1 ^c^
Triglycerides (mg/dL)	99 ± 16.2	155.3 ± 18.4 ^a^	125.7 ± 17.7	127.6 ± 20.3
Total Cholesterol (mg/dL)	183.3 ± 7.5	202.9 ± 7.5 ^a^	189.4 ± 2.4	197.8 ± 7.0
Subcutaneous fat (g)	1.09 ± 0.12	3.6 ± 0.34 ^a^	0.69 ± 0.09	1.24 ± 0.37 ^c^
Visceral fat (g)	1.11 ± 0.10	1.85 ± 0.12 ^a^	0.56 ± 0.09 ^a^	1.14 ± 0.2 ^bc^
Mesenteric (g)	0.47 ± 0.06	0.86 ± 0.07 ^a^	0.14 ± 0.04 ^a^	0.24 ± 0.07 ^c^
Liver (g)	1.65 ± 0.06	2.14 ± 0.17 ^a^	1.5 ± 0.08	1.55 ± 0.03 ^c^
Pancreas (g)	0.3 ± 0.02	0.25 ± 0.03	0.23 ± 0.02 ^a^	0.24 ± 0.02
Spleen (g)	0.09 ± 0.01	0.11 ± 0.02	0.09 ± 0.03	0.13 ± 0.02
Kidney (g)	0.55 ± 0.04	0.64 ± 0.04	0.48 ± 0.03	0.49 ± 0.02 ^c^

Data obtained from 4–8 samples and expressed as the mean ± standard error. ^a^ *p* < 0.05 versus WT-CD. ^b^ *p* < 0.05 versus HSP60Tg-CD. ^c^ *p* < 0.05 versus WT-HFD. CD: chow diet; HFD: high-fat diet; HSP60: heat shock protein 60; Tg: transgenic; WT: wild type.
